# Recent Advances in Membrane-Based Electrochemical Hydrogen Separation: A Review

**DOI:** 10.3390/membranes11020127

**Published:** 2021-02-13

**Authors:** Leandri Vermaak, Hein W. J. P. Neomagus, Dmitri G. Bessarabov

**Affiliations:** 1 HySA Infrastructure Centre of Competence, Faculty of Engineering, Potchefstroom Campus, North-West University, Potchefstroom 2520, South Africa; 2 Centre of Excellence in Carbon Based Fuels, Faculty of Engineering, Potchefstroom Campus, School of Chemical and Minerals Engineering, North-West University, Potchefstroom 2520, South Africa; Hein.Neomagus@nwu.ac.za

**Keywords:** electrochemical hydrogen separation, electrochemical hydrogen pump, proton exchange membrane (PEM), hydrogen purification/separation

## Abstract

In this paper an overview of commercial hydrogen separation technologies is given. These technologies are discussed and compared—with a detailed discussion on membrane-based technologies. An emerging and promising novel hydrogen separation technology, namely, electrochemical hydrogen separation (EHS) is reviewed in detail. EHS has many advantages over conventional separation systems (e.g., it is not energy intensive, it is environmentally-friendly with near-zero pollutants, it is known for its silent operation, and, the greatest advantage, simultaneous compression and purification can be achieved in a one-step operation). Therefore, the focus of this review is to survey open literature and research conducted to date on EHS. Current technological advances in the field of EHS that have been made are highlighted. In the conclusion, literature gaps and aspects of electrochemical hydrogen separation, that require further research, are also highlighted. Currently, the cost factor, lack of adequate understanding of the degradation mechanisms related to this technology, and the fact that certain aspects of this technology are as yet unexplored (e.g., simultaneous hydrogen separation and compression) all hinder its widespread application. In future research, some attention could be given to the aforementioned factors and emerging technologies, such as ceramic proton conductors and solid acids.

## 1. Introduction

The continued expansion of the commercial and industrial sectors, such as heavy-duty mobility/shipping and manufacturing, raises concerns regarding the supply capacity of existing energy resources [1,2]. Currently the global energy demand is primarily met by fossil fuel utilisation methods, which raises environmental concerns related to CO_2_ emissions and other greenhouse gas emissions [2,3]. Subsequently, the energy sector faces a major challenge—to decarbonise energy supply and to find new reliable, sustainable, and environmentally friendly energy alternatives [4,5]. Renewable energy (RE) is expected to play a key role in future energy systems as it is clean and sustainable [2]. However, some key challenges, such as its variable and intermittent nature [3,6,7] remain to be addressed before completely transitioning towards RE [2]. The solution to this problem lies in adequate large-scale energy storage, which would increase energy supply reliability [8]. Energy storage can provide energy flexibility and will reduce the global dependence on fossil fuel backup power. Various types of energy storage systems exist [6,9,10]. These can be broadly categorized [8] as electrochemical (batteries) [11], chemical (hydrogen systems: Fuel cells/electrolyses [12,13,14]), electrical (capacitors, super capacitors and ultra-capacitors) [15,16], mechanical (flywheels [17,18,19], compressed air [20,21] and pumped hydro-storage [22,23]) and thermal (hot water, sensible/latent heat storage, solar energy storage) [24,25,26,27,28], and magnetic (superconducting energy storage) [29]. Alternative methods for large-scale energy storage are being researched, including renewable hydrogen and synthetic natural gas [6].

Hydrogen is part of an industrial concept known as power-to-gas (P2G) technology, which is a power grid balancing mechanism used to capture and store surplus energy to use at times of limited supply (e.g., night-time or at times of low wind speed when solar and wind is used as energy source) [7]. In principle, P2G converts excess RE into a chemical carrier such as hydrogen or methane [7]. Hydrogen, in particular, is attracting great interest as an energy carrier, with many unique properties to commend itself [30,31]. It can be produced/converted into electricity by means of electrochemical devices (e.g., electrolysers and fuel cells) with relatively high energy conversion efficiencies and can be stored using a variety of methods [3,31,32,33,34,35], such as compressed gas, cryogenic liquid [36,37], chemical compounds (e.g., liquid organic hydrogen carriers (LOHC) [38,39,40], ammonia) [41] or it can be adsorbed/absorbed on special materials (e.g., metal hybrids [42], chemical hybrids, carbon nanostructures). Long-distance hydrogen transportation can be achieved through pipelines [43,44] or via tanker trucks [45], and can be converted into various forms of energy more efficiently than other fuels [30,31]. Furthermore, hydrogen can be generated in an environmentally friendly manner with no greenhouse gas pollutants [30,31]. Hydrogen also has potential to provide energy to the main sectors of the economy, including transportation, buildings, and industry [46,47]. This, in turn, may lead to a low-carbon energy system known as the “hydrogen economy”, which was introduced in 1972 [48].

Currently, hydrogen is a very important industrial commodity, as it is a key reactant and/or by-product of several industrial processes, including the food industry, petrochemical and petroleum refining, ammonia production, methanol production, hydrogenation processes, hydrometallurgical processes, and metal refining (mainly nickel, tungsten, molybdenum, copper, zinc, uranium, and lead) [3,46,49,50,51]. It can also be employed for application in electricity production from fuel cells, transportation, and energy storage [51]. 

Hydrogen is not widely available in gaseous state, but rather in a form of chemical compounds in natural sources, such as natural gas, water, coal and biomass (after gasification), which are all major feedstocks for hydrogen production [52]. Many hydrogen production pathways can be found in literature [45,53,54,55] and the selection thereof is mainly dependent on the feedstock used to produce hydrogen, the scale of production, and the available energy sources [2]. These pathways can be classified in various ways: The hydrogen source/feedstock (hydrocarbons or non-hydrocarbons) [2,3,56,57], the chemical nature and/or energy input [2,3,56] (thermochemical, electrochemical and biological [46]), the production method used [3,52,56,57] (its maturity level and efficiency) [2], the catalyst material [52], storage [51], the distribution mechanism (i.e., on-site generation or delivered) [53,56] and end use (e.g., hydrogen purity required) [50]. The choice of the hydrogen production pathway should take into account, (i) the hydrogen fuel quality grade required for end-use application and (ii) purification technology feasibility [2]. Separation processes, such as pressure swing adsorption (PSA), are applied to improve the economics of the conventional hydrogen production methods [58].

Table 1 summarizes the state-of-the-art hydrogen production technologies based on their advantages and disadvantaged, the technology maturity level (TML), the process efficiency, cleanness of the hydrogen, and the impurities commonly contained in the product streams [2].

For hydrogen production, fossil fuels are currently the main source [59]. Fossil fuel-based hydrogen production technologies are already developed and mature industrial technologies [60], capable of producing high grade hydrogen at relatively lower costs compared to some alternatives [59]. Therefore, of the over 50 million tons of hydrogen produced annually, fossil fuel-based hydrogen production constitutes an estimated 95% [3,61]. There are a number of feedstocks used to produce industrial hydrogen, but the most favoured feedstock is natural gas due to it being abundantly available and cost efficient [52,62]. 

The two main methods used in industry to produce hydrogen from fossil fuels are reforming processes and gasification [53]. These two methods are distinguished by the nature of the incoming fuel [3]. Gasification processes use solid fuel, such as coal, biomass and solid waste to produce hydrogen or syngas (a mixture of mainly H_2_, CO [63] and, in some instances, CO_2_ [46,64,65]), while reforming processes make use of fluid fuel, either in gas or liquid form, for syngas production [3]. Three reforming processes can be differentiated to produce hydrogen from hydrocarbons: (i) Steam reforming (particularly steam methane reforming (SMR)) [55,61,65,66,67,68], (ii) partial oxidation (POX) [3,46,66], and (iii) auto-thermal reforming (ATR) [3,55,65]. These processes are distinguished by the reactants involved and the thermodynamic nature of the reactions taking place [3,55]. For example, in SMR and steam-gasification, steam (water) reacts with hydrocarbons to produce hydrogen. This reaction is endothermic. In the case of POX and gasification, oxygen reacts with the hydrocarbons to produce hydrogen and results in an exothermic reaction. When these two reactions are combined (SMR and POX), the process is termed as ATR [3,55,67]. In addition to H_2_, CO_2_ and CO are emitted by reforming processes [46,65]. Other hydrogen reforming technologies can also be found in literature, such as hydrocarbon pyrolysis, plasma reforming, ammonia reforming and aqueous phase reforming [67,69]; however, they are not as common as SMR and coal gasification. In the majority of the processes listed above, CO_2_ and/or CO is produced. One of the promising technologies that receive significant attention is the utilization of CO_2_ by reacting it with H_2_ to produce valuable chemicals, such as methane and methanol (e.g., through the Sabatier process) [70,71,72,73]. Similarly, CO can be converted through the water–gas shift (WGS) reaction [3,55,60,67,74]. 

Although fossil fuels are currently the main feedstock used to produce hydrogen, renewable integrated technologies are unavoidable for the global energy future [67]. Several processes have been proposed for hydrogen production from renewables [87]. Though not widely implemented, hydrogen can be produced from biomass using processes such as pyrolysis/gasification, but this is commonly accompanied by large amounts of impurities [31,61,65,66,88]. Several methods of hydrogen production from water are also available, including electrolysis, thermochemical processes, photolysis, and direct thermal decomposition or thermolysis [61]. Water electrolysis is a common method used to produce hydrogen; furthermore, it is the only method, at present, that can be used for large-scale hydrogen production without fossil fuel utilization [31]. One major advantage of water electrolysis is that no-carbon containing compounds are present in the exhaust, only water [2,66,67]. Hydrogen is obtained by splitting water into oxygen and hydrogen, achieved by an electrical current [89]. The electricity required for electrolysis can be generated from renewable sources (e.g., solar, wind and hydropower) or non-renewable sources (fossil fuel or nuclear-based) [90]. 

The benefits of hydrogen as a fuel, which is clean and efficient, can only be fully recognized when hydrogen is produced from renewable energy sources [6]. Most of the current hydrogen production methods yield hydrogen-rich streams, but are commonly accompanied by contaminant gases including CO_2_, CO, sulphur-containing components, CH_4_, and N_2_ (see Table 1). Shalygin et al. [91] gives a detailed composition of all small to large-scale hydrogen production process streams. High-purity hydrogen (>99.97%) is required for fuel cells (according to SAE J2719—see Table 2), the chemical industry and stationary power production. The hydrogen produced from commercial processes should, therefore be purified after production, based on end-use application, e.g., fuel cells.

The purpose of this review is twofold: (i) To provide a general overview and comparison of the commercially available hydrogen separation technologies, and (ii) to survey open literature, on the status, and advances made, in the field of electrochemical hydrogen separation (EHS). With the former, special emphasis is given to membrane technologies, especially the membrane materials/types and their performance properties. With the latter, all available literature on EHS are summarized and classified based on the type of article (experimental, modelling, case study or review) and the year it has been published, the operating temperature range, the membrane materials, the type of electrocatalyst and the type of impurities contained in the feed. In the conclusion, literature gaps in the field of EHS is identified for further (future) research.

## 2. Hydrogen Separation/Purification Technologies 

For hydrogen to be realized as a widespread energy carrier (especially as a carrier of RE), its purification and compression are unavoidable industrial processes. Several technological approaches are used to extract hydrogen from gas mixtures, utilizing various characteristics of hydrogen, under different industrial conditions. Common approaches for hydrogen recovery include the following: Adsorbing the impurities (pressure swing adsorption, PSA), condensing the impurities (cryogenic distillation) or by using permselective membranes [49,92,93]. Although PSA and cryogenic distillation processes are both commercial processes used in hydrogen separation, pressure-driven membranes are considered a better candidate for hydrogen production because they are not as energy intensive and they yield high-purity hydrogen [92]. Moreover, PSA and cryogenic distillation technologies all require multiple units and, in some instances, may involve supplementary wash columns to remove CO and CO_2_ [49]. Additional advantages offered by membrane technologies include the ease of operation, low energy consumption, possibility of continuous operation, cost effectiveness, low maintenance and compactness [86,92,94,95,96]. Nonetheless, despite their numerous advantages, membrane systems commonly depend on high-pressure feed streams and hydrogen embrittlement is often experienced [49]. As a rule, hydrogen appears in the permeate (low pressure stream after membrane), and additional compression is required after hydrogen purification for transport and storage purposes [97]—including expenses of energy, additional equipment, etc. Properties of the various hydrogen purification technologies are summarized in Table 3. A brief discussion of each technology then follows. 

### 2.1. Pressure Swing Adsorption 

PSA is the most extensively used state-of-the-art industrial process for hydrogen separation [100]; it is capable of yielding hydrogen with a purity ranging from 98–99.999% [2,81,97]. It is most frequently used in the chemical/petrochemical industry, as well as to recover hydrogen from industrial-rich exhaust gases, including reforming off-gases, coke oven gases, and pyrolysis effluent gases [92,94,101]. Currently, about 85% of the produced hydrogen is purified by PSA [102]. Although the system is mainly classified as a batch system, continuous operation can be achieved by implementing multiple adsorbers [92,93,94], creating a cyclic process [103,104]. The system can be divided into five primary steps: (i) Adsorption, (ii) concurrent depressurization, (iii) counter-current depressurization, (iv) purge and (v) counter-current pressurization [94,100]. 

In PSA, a hydrogen-rich gas mixture is passed through a high-surface-area adsorber, capable of adsorbing the impurities (e.g., CO, CO_2_, CH_4_, H_2_O and N_2_ [105]), whilst allowing hydrogen to permeate through the material [103]. The impurities are removed by swinging the system pressure from the feed to the exhaust pressure, coupled with a high-purity hydrogen purge. The driving force of PSA is the difference in the impurities’ gases’ partial pressure of the feed gas and the exhaust. Generally, hydrogen separation requires a pressure ratio of 4:1 between the feed and the exhaust [103]. In the initial layer H_2_O, CO_2_ and CH_4_ is removed, whilst a second layer removes other components until the levels of CO is <10 ppm [105]. The reaction takes place at room temperature and at pressures of 20–25 bar [104]. Zeolites are commonly used as adsorbent materials [103]. To increase the hydrogen recovery, a complex arrangement of the columns is required; generally, more than eight columns are required [105]. The quantity of recovered hydrogen is dependent on the feed and purge gas pressures and hydrogen-to-impurity ratio [92]. The hydrogen recovery is typically in the range between 60% and 90% [97]. 

### 2.2. Cryogenic Distillation 

Cryogenic distillation is a widely used separation process at low temperature (LT). It is used to separate gas components based on differences in their boiling temperatures [92,93,94]. Hydrogen’s low boiling point of −252.9 °C (below that of almost all other substances) is used as a measure to separate it from other components, where the collected hydrogen can be stored as a liquid [106]. The gas needs to be cooled down, to condense, resulting in large energy consumption [92,94].

If significant amounts of CO, CO_2_ and N_2_ are found in the feed stream, a methane wash column is required to reduce the concentrations of these gases [92,93]. The feed gas also requires pretreatment to remove the components that might freeze; therefore, water should be reduced to <1 ppm and CO_2_ to <100 ppm [94]. It is not practical to use this method to obtain high-purity hydrogen, however, higher hydrogen recovery can be achieved at moderate hydrogen purity yields (≤95%) [92,94]. Similar to PSA, cryogenic distillation is perfect for large industrial scales, but unsuitable for small portable applications [103].

### 2.3. Membrane Technologies 

**Figure 1:**
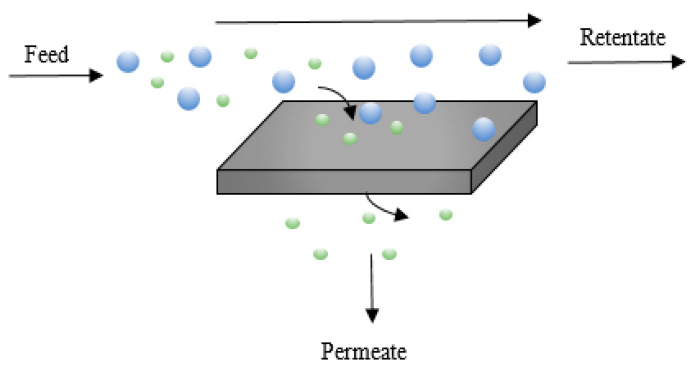
Simplified schematic of membrane separation.

Besides PSA and cryogenic distillation, membrane separation has attracted the widest interest. A membrane is a selective barrier between two phases [107] that allows mass transfer under the action of a driving force [108] (e.g., gradients in pressure, temperature, concentration or electrical potential [107]). This allows for preferential permeation of some components of the feed stream, with retention of the other components [109]. See Figure 1. 

**Figure 2:**
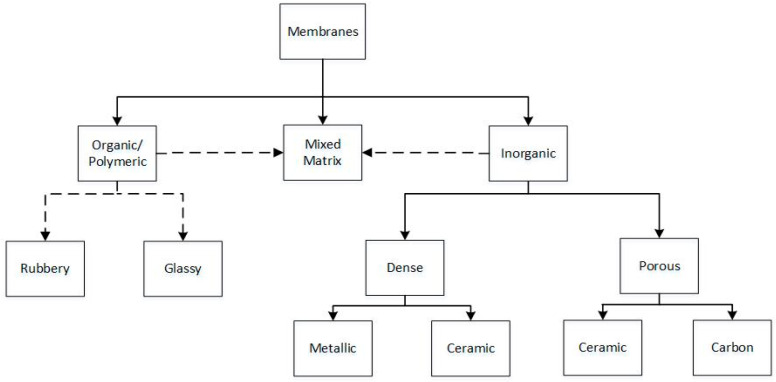
Schematic of membrane classification.

Membranes for hydrogen separation can be divided into organic (polymeric), inorganic and mixed-matrix (hybrid). See membrane classification scheme in Figure 2, which is based on the nature of the material of the membranes. Currently, industrial processes mainly use polymer membranes (glassy or rubbery [110]) due to their capability to cope with high pressure drops, their low cost and good scalability [108]. Though organic/polymeric membranes are temperature limited (363–373 K) [108,111], recent progress have been made with thermally rearranged polymers—which show good separation at high temperatures [112,113]. Inorganic membranes provide several advantages, including mechanical, thermal and chemical stability [114], it’s typically not subject to dimensional changes, such as plasticization, or swelling of the membrane upon adsorption of the components of the feed gas and controllable pore size distribution allowing for better control over selectivity and permeability [108]. To take advantage of the capabilities of inorganic membranes combined with the low manufacturing costs of the organic membranes, mixed-matrix membranes have been developed (composite materials that consist of continuous polymeric matrix and imbedded, mainly inorganic, particles) [115]. According to their characteristics, membranes can be classified as either dense (non-porous) or porous [92,96]. Porous membranes can be divided into glasses, organic polymer, ceramic- and carbon-based membranes (carbon molecular sieves) [116]. A particular important type of membranes is metallic or metal-alloys membranes (mainly Pd and/or its alloys [110]). Ceramic proton-conducting membranes are also known, but are still at the earlier development stage [108]. According to the IUPAC classification of pores sizes, porous membranes can be either microporous (d_p_ < 2 nm), mesoporous (2< d_p_ < 50 nm), or macroporous (d_p_ > 50 nm), where d_p_ is the average pore diameter [108].

**Figure 3:**
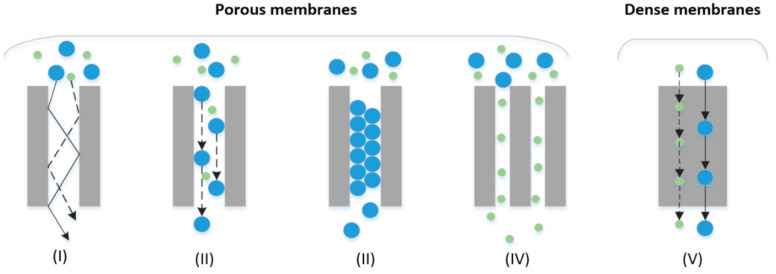
Transport mechanism for porous membranes ((**I**) Knudson diffusion, (**II**) surface diffusion, (**III**) capillary condensation, and (**IV**) molecular sieving) and dense membranes ((**V**) solution–diffusion).

Gas transport through membranes can occur through a number of mechanisms [109]. A comprehensive review of the permeation mechanisms of inorganic membranes can be found in Oyama et al. [117]. Porous membranes achieve fractionation based on differences in size, shape and/or affinity between the permeating molecules and the membrane [108]. Depending on the pore size, different mechanisms will dominate molecular transport [118]. Mechanisms associated with porous membranes include, broadly: (i) Knudsen diffusion, (ii) surface diffusion, (iii) capillary condensation and (iv) molecular sieving (MS) [79,92,103,109,119]. See Figure 3. MS is an activated process that will be dominant for the smallest pores (pore diameter and diameter of diffusing molecules are approximately the same). Whereas surface diffusion will dominate the some somewhat larger pores, and Knudsen diffusion will dominate the even larger pores [118]. Separation based on Knudsen diffusion is mainly determined by the pore size and it occurs when the pore diameter of the membrane is smaller than the mean free path of the gas being separated [107]. Separation based on MS operate on size-exclusion principal [92]. In other words, only molecules with small enough kinetic diameters can permeate through the membrane [103]. Separation based on capillary condensation takes place when a partially condensed gas phase occupies a pore. Only gases that are soluble in this condensed phase can permeate through the pores when the pores are completely filled [103]. Surface diffusion can occur alongside Knudson diffusion. It involves the adsorption of gas molecules onto the pore walls of the membrane and spread along the surface [92,103]. Permeability will be high for the molecules that are readily adsorbed onto the pore walls. However, this form of diffusion is limited to certain temperatures and pore diameters [103]. In the case of dense membranes, molecular transport occurs through a solution–diffusion (Sol–D) mechanism [120]. Thus, for the separation of hydrogen from other components in a gas mixture, both size (diffusivity) and the condensability (solubility) of the gases to be separated play important roles [92,108,111,120,121]. 

Hydrogen purification typically involves the separation of H_2_ from light gas molecules, such as CO_2_, CO, CH_4_, H_2_O and impurities, e.g., H_2_S. Physical adsorption becomes negligible at temperatures >400 °C, therefore hydrogen separation is mainly based on MS (hydrogen kinetic diameter = 2.89 Å) and differences in molecular diffusivity [114,122]. Gas transport through non-porous metals (Pd) involves dissociation of H_2_ molecules into atomic hydrogen and its transport through the films [123]. Knudsen diffusion or a combination of Knudsen diffusion and surface diffusion are characteristic for porous metal membranes, e.g., porous stainless steel [124,125]. Likewise, hydrogen transport through ceramic membranes is based on Sol–D (dense ceramic) and MS (microporous). Gas transport in polymeric membranes and carbon membranes, on the other hand, occurs based on the Sol–D mechanism and MS, respectively. The kinetic diameters of light gases commonly found in produced H_2_ streams, such as He, H_2_, NO, CO_2_, Ar, O_2_, N_2_, CO and CH_4_, can be found in Teplyakov and Meares [126] and Nenoff et al. [127].

Two basic properties are used to evaluate the performance of a membrane: Permeance (also commonly referred to as the flux or permeation rate [107]) and selectivity [92,108,109,111]. The higher the selectivity, the lower the driving force required to achieve a certain separation—thereby reducing the cost of operation of the system. Conversely, the higher the flux, the smaller the required membrane area—thereby reducing the capital cost of the system [111]. Permeance is defined as the net transport of constituents through the membrane and it can be expressed as either mass per unit time and unit area, or mole per unit time and unit area [79]. It is usually used to assess the permeability for composite and asymmetric membranes [109]. The permeance in mol·m^−2^·s^−1^·Pa^−1^ (SI) (or cm^3^(STP)·cm^−2^·s^−1^·cm^−1^ Hg)), is the permeability coefficient *P_i_* divided by the membrane thickness (cm, or sometimes reported in m) [92,117,128]:

(1) $$Q_{i}=\frac{P_{i}}{\delta }$$

Permeability coefficient, or permeability, is typically used to define the membrane’s capacity to perform gas transport. In other words, permeability is the measure of the ability of certain gas components to diffuse through a membrane [103], where high permeability indicates a high throughput [111]. It denotes the amount of hydrogen (molar/volume) diffusing through the membrane per unit area and time at a given pressure gradient [103,111]. Several units are commonly used for permeability, including Barrer (10^−10^ cm^3^(STP)·cm·cm^−2^·s^−1^·cm^−1^ Hg = 3.347 × 10^−16^ mol·m^−1^·s^−1^·Pa^−1^ (SI units)), gas permeability units (GPU = 10^−6^ cm^3^(STP)·cm^−2^·s^−1^·cm^−1^ Hg) or molar permeability (mol·m·m^−2^·s^−1^·Pa^−1^) for characterization of the permeance of membranes. 

The selectivity of a membrane measures the membrane’s ability to separate a desired component from the feed mixture [108]. Membrane selectivity towards gas mixtures and mixtures of organic liquids is usually expressed by a separation factor *α*. The ideal selectivity/permselectivity [111] is defined as the permeability or permeance ratio of two pure gases:

(2) $$\alpha_{i/j}=\frac{P_{i}}{P_{j}}=\frac{Q_{i}}{Q_{j}}$$

where, *P_i_* and *P_j_*, and *Q_i_* and *Q_j_* are the permeability coefficients and permeance as gases *i* and *j*, respectively. 

Typically, the more permeable gas is taken as *i*, so that *α_i/j_* >1 [129]. Ideal selectivity is very useful for the description of the separation of mixtures of light gases, such as H_2_ and N_2_, which have low solubility in membrane materials. Thus, the gases only weakly affect the property and behaviour of the polymer, they do not affect the mutual diffusion and sorption parameters in the process of simultaneous transport of gases in mixture separation [128]. Whereas, in the case of heavy vapours/gasses with great solubility, the applicability of this parameter is not as predictable [128]. The following equation is used for mixture separation [128]:

(3) $$\alpha_{i/j}{}^{*}=\frac{y_{i}/y_{j}}{x_{i}/x_{j}}$$

where, *y_i_* and *y_j_* depict the concentration of components i and j in the permeate. Mass and molar concentrations (kg.m^−3^; mol·m^−3^), as well as weight fraction, mole fraction, and volume fraction are frequently used. As for *x_i_* and *x_j_*, two definitions are found in literature and are considered. According to Koros et al. [130], if *x_i_* and *x_j_* characterize the composition of the feed stream, this ratio (Equation (3) is termed the *separation coefficient*, similarly, if *x_i_* and *x_j_* refer to the composition in the retentate, the ratio is termed the *separation factor* [128]. The former is also sometimes referred to as the ideal/“actual” selectivity [107]. If *α_A/B_ = α_B/A_* = 1, no separation is achieved [107]. In the case where the feed pressure is significantly larger than the permeate pressure, and the permeate pressure approaches zero, the ideal selectivity and real selectivity will be in equal [109]. 

A fundamental expression for transport in membranes is derived from Fick’s first law, which relates the flux of species i to the concentration gradient. Under steady state conditions, this equation can be integrated to give:

(4) $$J=-D\frac{dC}{dx}$$

(5) $$J_{i}=\frac{D_{i}\left(C_{i,0}-C_{i,\delta }\right)}{\delta }$$

where, *J_i_* is the flux (cm^3^(STP)·cm^−2^·s^−1^ or mol·m^−2^·s^−1^), *D_i_* is the diffusion coefficient (m^2^·s^−1^), *C*_*i*,0_ and *C_i,δ_* depicts the inlet and outlet concentrations (usually mol·m^3^), and *δ* is the membrane thickness (m) [98,131]. 

#### 2.3.1. Porous Membranes 

The transport mechanism for each mechanism (porous membranes) can be summarized as follows [118,132]. The molar flux through the pores can be defined by Fick’s law Equation (4). According to Burggraaf et al. [133] and Bhandarkar et al. [134], the Fickian diffusion coefficient (*D_i_*) is composed of a corrected diffusion coefficient together with a thermodynamic correction factor *Γ*(*T,P*):

(6) $$D_{i}=D_{i,c}\Gamma ,\;\mathrm{where}\;\Gamma \equiv \frac{\partial lnp}{\partial lnC}$$

The diffusion coefficients are defined as:

(7) $$D_{K,c}=\frac{d_{p}}{3}\sqrt{\frac{8RT}{\pi M}}\;(\mathrm{Knudsen})$$

(8) $$D_{S,c}=D_{S}^{0}exp\left(\frac{-E_{a,S}}{RT}\right)\;(\mathrm{Surface}\;\mathrm{diffusion})$$

(9) $$D_{MS,c}=D_{MS}^{0}exp\left(\frac{-E_{a,MS}}{RT}\right)\;(\mathrm{Molecular}\;\mathrm{sieving})$$

where, *E_a_* denotes the activation energy (kJ·mol^−1^), R is the universal gas constant and *T* is the temperature (*K*). Different methods for obtaining *D_K,c_* have been reported [98,117].

If ideal gas behaviour is assumed, *Γ* = 1 and Equations (4) and (7) can be combined and integrated to give:

(10) $$J_{K}=\frac{D_{K}\Delta p}{RT\delta }$$

where, *δ* is the thickness of the membrane and Δp is the difference in the outlet and inlet partial pressures. Similarly, the integrated flux equations for activated processes, assuming Henry’s law for adsorption, can be written as [132,133,135]:

(11) $$J_{i}=\frac{\Delta p}{RT\delta }D_{0}\left(T\right)exp\left\{\frac{-\left(E_{a}-E_{ads}\right)}{RT}\right\}$$

where, *E_ads_* denotes the adsorption energy (kJ·mol^−1^). 

Substituting these expressions into the flux equation and defining the permeability, *Pi*, and permeance, *Qi*, due to a particular transport mechanism as $Q_{i}=\frac{P_{i}}{\delta }=\frac{J_{i}}{\Delta p}$ the following temperature dependencies for the permeance due to the transport mechanism are obtained:

(12) $$R_{K}∼{\left(MRT\right)}^{-0.5}\;(\mathrm{Knudsen})$$

(13) $$R_{S}∼D_{0}\left(T\right)exp\left(\frac{-\left(E_{a,S}-E_{ads}\right)}{RT}\right)\;(\mathrm{Surface}\;\mathrm{diffusion})$$

(14) $$R_{MS}∼D_{0}\left(T\right)exp\left(\frac{-\left(E_{a,MS}-E_{ads}\right)}{RT}\right)\;(\mathrm{Molecular}\;\mathrm{sieving})$$

In the case of Knudsen diffusion, the selectivity can be calculated using Equation (15) [111]:

(15) $$\alpha_{i/j}=\sqrt{\frac{M_{j}}{M_{i}}}$$

#### 2.3.2. Dense (Non-Porous) Membranes-Diffusion Mechanism

Solid-state diffusion occurs with a further decrease in the pore size, where the gas molecules interact strongly with the membrane material and its solubility needs to be considered [117]. In this case, the permeability can be determined using the equation that is based on the sol-D model [103,107]:

(16) P=D×S

where, *S* is the gas solubility in mol·m^−3^·Pa^−1^ (see Equation (23)). If Equation (16) is substituted into Equation (2), it is possible to speak of selectivity of diffusion and sorption [128]:

(17) $$\alpha_{i/j}=\left(D_{i}/D_{j}\right)\left(S_{i}/S_{j}\right)$$

There are three instances where this transport mechanism applies: (i) permeation through glassy membranes, (ii) metallic membranes, and (iii) polymeric membranes [117,136]. Overall, the permeability can be calculated using the following expression [98,128]:

(18) $$P_{i}=\frac{J_{i}\delta }{\Delta p}$$

Steady-state flux of gases through a dense (non-porous) metallic membranes can be expressed by Equation (1) [137], which is derived by combining Fick’s first law (driving force = concentration gradient) (Equation (4) [107,138] and Henry’s law (Equation (20) [139]), as follows:

(19) $$\mathrm{Steady}\;\mathrm{state}\;\mathrm{permeability}:\;\;\;\;\;\;\;\;\;\;\;\;\;\;\;J_{i}=\frac{P_{i}\left(p_{i,feed}^{n}-p_{i,perm}^{n}\right)}{\delta }$$

(20) $$\mathrm{Henry}’s\;\mathrm{law}:\;\;\;\;\;\;\;\;\;\;\;\;\;\;\;\;\;\;\;\;\;\;\;\;\;S_{i}=\frac{C_{i}}{p_{i}^{n}}$$

where, *J_i_* is the flow rate (mol·m^−2^) of the diffusing species, *P_i_* is the permeability coefficient (mol·m^−1^·s^−1^·Pa^−1^) of component *i*, *δ* is the thickness of the membrane (*m*), and $p_{i,feed}^{n}$ and $p_{i,perm}^{n}$ are the hydrogen partial pressures at the feed and permeate side, respectively. Furthermore, *S_i_* and *C_i_*, refers to the solubility coefficient (mol·m^−3^·Pa^−1^), and the concentration gradient across the membrane, respectively. The pressure component (*n*) is generally in the range of 0.5–1, depending on the limiting step of the hydrogen permeation mechanism [108,117,139]. The hydrogen permeation is controlled by the rates of adsorption/desorption and the diffusion through the lattice [117,140]. Generally, if diffusion through the metal is the rate limiting step, the pressure is relatively low, and the hydrogen atoms form an ideal solution in the metal, then *n* = 0.5 known as Sievert’s law [108,111]. Higher values of *n* are expected when mass transport to/from the surface or dissociative/associative adsorption steps become rate determining [108]. Moreover *n* > 0.5 for defective membranes (e.g., pinhole formation) or when the rate is influenced by the membrane’s porous support [108]. Furthermore, the thickness of the membrane can also influence the value of *n*, causing it to be >0.5 [141]. 

The relationship between the permeability and temperature follows Arrhenius behaviour [142]:

(21) $$P=P_{0}exp\left(\frac{-E_{a}}{RT}\right)$$

where, $P_{i}^{0}$ is the maximum permeability at infinitely high temperature, $E_{a}$ is the activation energy for permeation, *R* is the universal gas constant, and *T* the absolute temperature. Similarly, the temperature dependence of the gas diffusion coefficient and solution coefficient can be expressed as follows (Equations (22) and (23)) [121]:

(22) $$D=D_{0}exp\left(\frac{-E_{d}}{RT}\right)$$

(23) $$S=S_{0}exp\left(\frac{-\Delta H_{s}}{RT}\right)$$

where, *E_d_* is the activation energy of diffusion, *ΔH_s_* is the partial molar sorption, *D*_0_ and *S*_0_ are the diffusivity and solubility, respectively, at infinite temperature [103]. Consequently, when *n* is 0.5, the flux can be written in terms of the so-called Richardson equation, where Equation (21) is substituted into Equation (19), as follows:

(24) $$J_{i}=P_{0}exp\left(\frac{-E_{a}}{RT}\right)\frac{\left(p_{i,feed}^{n}-p_{i,perm}^{n}\right)}{\delta }$$

#### 2.3.3. Ceramic Proton-Conducting Membranes 

Although proton-conducting ceramics are still at the early stages of development, several research efforts have been made [143,144,145,146,147,148,149,150]. Overall, the process of hydrogen permeation through a dense proton conducting membrane involves several steps [122,151]:

1. *H*_2_ gas diffusion to reaction sites on the surface of the feed side;
2. *H*_2_ adsorption, dissociation, and charge transfer at the membrane surface;
  $$H_{2}\to H_{2,ads}\;;\;H_{2,ads}\to 2H_{ads};\;H_{ads}\to H^{+}+e^{-}$$
3. Proton reduction and hydrogen re-association at the membrane surface
  $${\left(H_{ads}^{+}+e^{-}\right)}_{S^{\prime }}\overset{\;incorporation\;}{\to }{\left(H_{ads}^{+}+e^{-}\right)}_{BM}\overset{\;diffusion\;}{\to }{\left(H_{ads}^{+}+e^{-}\right)}_{S^{\prime \prime }}\overset{\;re-association\;}{\to }{\left(H_{2}\right)}_{S^{\prime \prime }}\overset{\;diffusion\;}{\to }{\left(H_{2}\right)}_{G}$$
  where, *S’*, *BM*, *S”* and *G* is the membrane surface at the inlet, the bulk membrane, the membrane surface at the outlet, and the gas, respectively.

Two transport mechanisms have been proposed in literature: The vehicle mechanism and the Grotthus/proton hopping mechanism [151]. In the vehicle mechanism, protons bind to oxygen to form a hydroxide ion which diffuses through the lattice by vacancy or interstitial diffusion. Electroneutrality is achieved through the counter-diffusion of unprotonated vehicles or oxygen vacancies [108]. The Grotthuss mechanism instead assumes that protons jump between stationary oxygen ions, and that the diffusion of protons and electrons occur in the same direction, whilst maintaining electroneutrality and a zero net electric current [151,152]. For structural reasons, proton transport in oxides is better explained by the Grotthuss mechanism [153].

The proton flux (mol·cm^−2^·s^−^^1^) for a membrane containing only protonic-electronic conductors can be described by the Wagner equation [108,154]:

(25) $$J_{H^{+}}=-\frac{RT}{2F^{2}\delta }\int_{I}^{II}\left(\sigma_{H^{+}}\times t_{e^{-}}\right)dlnp_{H_{2}}$$

where, *R* is the universal gas constant (8.314 J·mol^−1^·K^−1^), *T*(*K*) is the absolute temperature, *F* is the Faraday constant (96 485 C·mol^−1^), *δ* is the thickness of the ceramic membrane (*m*), $\sigma_{H^{+}}$ (S·cm^−1^) is the proton conductivity within the membrane, $t_{e^{-}}$ is the electronic transport number, and $p_{H_{2}}$ is the hydrogen partial pressure (*Pa*). In this equation, the pressure gradient serves as driving force and the flux is directed from *I*→II [108]. Since $\sigma_{H^{+}}$ and $t_{e^{-}}$ can be dependent on the pressure, Norby and Haugsrud [152] and Marie-Laure et al. [153] integrated Equation (20) for different limiting cases. For example, when $t_{e^{-}}≅1$ and $\sigma_{H^{+}}$ is proportional to $p_{H_{2}}^{n}$ where (i) *n* = 0.5 when protons are minority defects, (ii) *n* = 0.25 when protons are majority defects compensated by electrons, and (iii) *n* = 0 when protons are majority defects compensated by acceptor dopants. In (i) and (ii), $J_{H^{+}}\sim \left[{\left(p_{H_{2}}^{n}\right)}^{I}-{\left(p_{H_{2}}^{n}\right)}^{II}\right]$ and, therefore, the partial pressure on the feed side (I) has a strong effect on the proton flux. In case (iii), $J_{H_{2}}\sim \left(p_{H_{2}}^{II}/p_{H_{2}}^{I}\right)$, and consequently the pressure on both sides are equally important [108,152].

A comparison of membrane properties, such as the temperature range, hydrogen selectivity and flux, and stability and poisoning issues are summarized in refs [79,92,103,155]. A more detailed quantification of hydrogen flux and selectivity for polymeric membranes can also be found in the following references [79,91,92,103,128,130,155]. Dense metal membranes (mainly palladium alloys) are seen as the most appropriate construction materials due to their high hydrogen selectivity [92,94,123,155]. However, Pt- and Pd-based membranes are known to have a high sensitivity to a variety of surface contaminants, such as H_2_S, CO, thiophene, chlorine and iodine [79]. These contaminants severely influence the performance of these membranes [79]. This is explained by the favourable interaction between the membrane and the contaminants. Membrane degradation/hydrogen embrittlement is also a common issue in metal membranes with high diffusivity or solubility (e.g., Pd-membranes) [140], which make them less durable. However, the problem of hydrogen embrittlement in Pd-based can be minimized by alloying the membranes [140] or by controlling the operating conditions to avoid a two-phased region [156]. Besides dense metallic membranes, dense ceramic membranes are also seen as a favourable option for hydrogen separation, for the same reason [155].

## 3. Electrochemical Hydrogen Separation 

Electrochemical membranes are seen as a promising alternative to pressure-driven membranes [94]. Electrochemical membranes are known to generate electricity (fuel cells) or to apply it (water electrolysis), and they are also used to purify/enrich and compress hydrogen streams [94]. EHS has the following advantages over other conventional separation systems [157,158]:

- Hydrogen, in the form of protons, is selectively transferred through the proton-conducting electrolyte;
- one-step operation provides pure hydrogen;
- the hydrogen separation rate can be controlled by the current (Faraday’s Law);
- a high hydrogen collection rate is achieved;
- simultaneous purification and compression of hydrogen is possible, in principle;
- high hydrogen separation is achieved at low cell voltages, with a high separation efficiency [156] and
- high selectivity and low permeability results in pure hydrogen (up to 99.99 vol.%) [159].

An additional benefit of this method is that CO_2_ is concentrated, and it can be captured and stored, without any further treatment—hence reducing greenhouse gas emissions [156,160]. In terms of application, electrochemical hydrogen separation could be beneficial in numerous industrial fields, including: (i) Hydrogen purification, for the use in fuel cell technologies, (ii) cooling agent in turbines, (iii) for the application in nuclear reactors (separation and concentration of hydrogen isotopes) and (iv) separation of hydrogen from natural gas mixtures (e.g., transportation of hythane through gas pipelines) [161]. Furthermore, compressed high-purity hydrogen is required for fuel cells (>99.97%) (SAE International, 2015) and hydrogen mobility (e.g., Hydrogen Mobility Europe (H2ME) [162,163]). 

### 3.1. Working Principle

**Figure 4:**
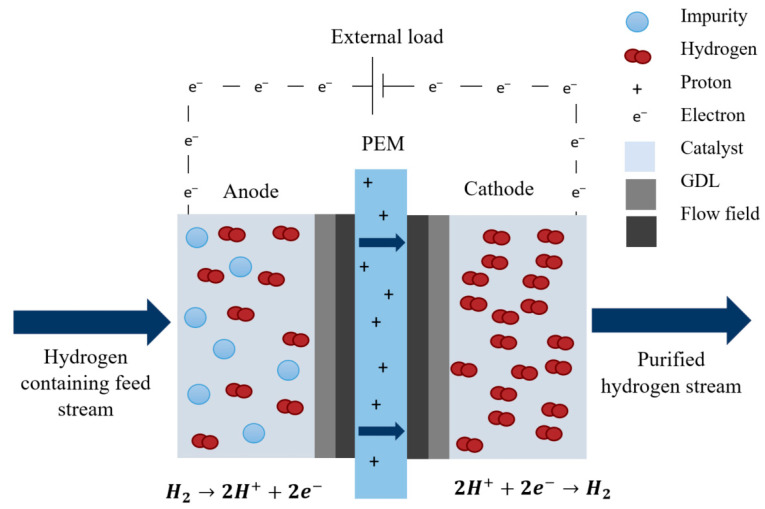
Schematic of the working principle of an ideal electrochemical hydrogen separator.

In principal, EHS is based on the following process (Figure 4). Molecular gaseous hydrogen (or a gas hydrogen-containing gas mixture) is injected at low pressure at the anode side of the proton exchange membrane (PEM) electrochemical cell. When the hydrogen makes contact with the anode electrode, hydrogen is oxidized into its constituents (protons and electrons), achieved with a Pt-based catalyst (Equation (26). The electrons then travel through an electrical circuit, whilst the protons move through the PEM to the cathode compartment. Finally, the protons and electron reduce to H_2_, at the cathode compartment (Equation (27). This can be carried out at high pressure to improve the specific volumetric energy density, for storage.

(26) $$\mathrm{Anode}\;\mathrm{reaction}:\;\;\;\;\;\;\;\;\;H_{2}\;\to \;2H^{+}+2e^{-}$$

(27) $$\mathrm{Cathode}\;\mathrm{reaction}:\;\;\;\;\;\;\;\;\;2H^{+}+2e^{-}\to \;H_{2}$$

(28) $$\mathrm{Overall}\;\mathrm{reaction}:\;\;\;\;\;\;\;\;H_{2}\left(Anode\right)\to \;H_{2}\left(Cathode\right)$$

In the case of hydrogen purification and separation, a hydrogen-rich gas enters the anode compartment and hydrogen is selectively transferred through the PEM. This reaction does not occur spontaneously. An external voltage (DC power source) is required to drive the chemical reaction (electrolytic mode) [164]. According to literature, minimal power is required to operate the cell [49]. The oxidation and reduction reactions of hydrogen are facile and corresponds to Nernstian/Faradic theoretical values in their electrochemical behaviour [49].

Unlike conventional membrane systems that rely on pressure or concentration differentials, the hydrogen permeation of an electrochemical hydrogen separator is only dependent on the applied current as driving force, which is quantified by the law of Faraday:

(29) $$I=nF\dot{n}$$

where, $\dot{n}$ depicts the flow of hydrogen (mol·s^−1^), I is the current (A), n depicts the number of electrons, and F is Faraday’s constant (96, 485 C·mol^−1^). Faraday’s law can also be rewritten in terms of the hydrogen mass flow rate ($\dot{m}$), which is proportional to the current [165]:

(30) $$\dot{m}=\;\frac{MI}{nF}$$

where, *M* is the molecular weight in kg·mol^−1^.

Although the cell overvoltage in this process is normally quite low, high hydrogen separation efficiencies can be achieved [156]. The purity of the hydrogen that is produced at the cathode side is high. This is highly dependent on several factors, for example: (i) The permeability of the membrane with respect to the feed gas composition, (ii) the integrity of the membrane and (iii) in the case of LT gas separation (<100 °C), when Nafion is used, the water content of the membrane [49].

### 3.2. Current Status of Electrochemical Hydrogen Separation Technology

A summary of previously reported literature on electrochemical hydrogen separation technologies is presented in Table 4 (in chronological order). The type of article, membrane, and electro-catalyst, as well as the gas mixtures that was used in the listed articles are included. A brief discussion on each contribution follows. The articles are characterized as high-temperature and low-temperature, with subsections “active” and “passive” gas mixtures. “Active” gas mixtures in this paper refer to gases that have affinity to either the membrane or the catalyst, e.g., CO and Pt catalyst. “Passive” gasses refer to gas mixtures that do not react with the membrane or the catalyst, typically made of permanent gases, such as N_2_ and Ar.

#### 3.2.1. Low-Temperature EHS 

##### “Passive” Gas Mixtures

- **H_2_/CH_4_ Mixtures**

Ibeh et al. [168] investigated hydrogen separation from H_2_/CH_4_ mixtures with both Pt and Pt-Ru catalysts. Results indicated that little energy was consumed and CH_4_ seemed to be inert at LTs, hence resulting in negligible fouling of the anode electrocatalyst. Moreover, it was found that if the hydrogen recovery is <80%, a small portion of the hydrogen energy is required for separation. A mathematical model was developed to simulate the electrochemical separation process. The model was adapted from the model used by Zhang [189]. This model takes into account the hydrogen adsorption and oxidation reaction, together with their reaction rates. Also, differential equations were used and solved using the ODE solver in Scilab. The differential equation included: The time dependence of the hydrogen surface coverage, the material balance for species *i*, the variation of the hydrogen pressure in the anode chamber, and the time dependence of the anode potential. There was a good agreement between the experimental data recorded and the simulated results.

- **H_2_/Ar Mixtures**

Nguyen et al. [159] used electrochemical impedance spectroscopy (EIS) to characterise and measure the kinetics and efficiency of the PEM and electrochemical cell. The roles of the cell structure and the different operating parameters (cell temperature, relative humidity and the partial pressure of hydrogen) on the overall process efficiency were investigated. Both the cell conductivity and electrode activity were measured. The relative humidity played a key role in the performance of the cell; the best performance (lowest membrane electrode assembly (MEA) resistance) were recorded when the cell and gas humidifier temperatures were similar, and close to 70 °C. Under these conditions, catalytic layers showed the highest activity. Consequently, lower catalyst activity was recorded at lower temperatures. Furthermore, when the humidifier and cell temperature difference was >10 °C, the water content for membrane humidification decreased, resulting in high MEA resistance. Moreover, mass-transport limitations were observed when the hydrogen partial pressure of the feed decreased. They concluded that an electrochemical hydrogen pump is limited to the treatment of gases in which the hydrogen content is >50%.

- **H_2_/N_2_ Mixtures**

Casati et al. [170] investigated the concentration of hydrogen achieved electrochemically from a lean hydrogen-inert gas mixture under galvanostatic and potentiostatic conditions. Results of the experimental runs suggested that galvanostatic operating conditions were unstable, whereas operation at potentiostatic conditions appears to be more stable. They reported that the hydrogen recovery increased with applied potential and the coefficient of performance (defined as the ratio of the hydrogen produced over the hydrogen consumed) decreased with the applied potential difference. Furthermore, the feed flow rate had a noteworthy effect on the recovered hydrogen. They proposed an optimum energy efficiency for separation, but did not identify its dependency on the process parameters.

The current efficiencies for current densities between 0 and 2 A·cm^−2^ were >83% and >90% for current densities ≥0.4 A·cm^−2^. Premixed natural gas reformate (35.8% H_2_, 1906 ppm CO, and 11.9% CO_2_, with the balance N_2_) and methanol reformate (1.03% CO and 29.8% CO_2_, with the balance H_2_) were used to investigate the CO tolerance and the cells’ tolerance to impurities. Near Faradic flows were achieved, irrespective of the feed composition. The effect of CO on the Pt catalyst was completely reversible at 120–160 °C. Furthermore, the CO concentrations were reduced from 1906 ppm to ~12 ppm, and CO_2_ concentrations were reduced from 11.9% to 0.37% at 0.4 A·cm^−2^ and 0.19% at 0.8 A·cm^−2^. Results of these experiments justified the use of polybenzimidazole (PBI) membrane-based hydrogen purification at high-temperature (HT) from feed streams containing relatively high CO and CO_2_ concentrations and low concentrations of hydrogen—not observed at LT (<100 °C), due to catalyst poisoning and water management issues.

Grigoriev et al. [161] investigated the extraction and compression of hydrogen from a H_2_/N_2_ mixture. They found that a lower hydrogen content in mixtures resulted in high mass transport limitations and that the compression efficiency decreased as the hydrogen content decreased. The current density of the compressor must be reduced in order to match mass transport limitations that result from lower hydrogen concentrations in the feed, in order to avoid low concentration and compression efficiencies. They concluded that this system could not be used to effectively extract hydrogen from diluted hydrogen streams. 

Schorer et al. [97] reports on the MEMPHYS (Membrane based purification of hydrogen system) project (including the project itself, project targets, and different work stages). Early measurements have been conducted and the results are discussed. The system was able to extract H_2_ from H_2_/N_2_ gas mixtures (1:1 and 4:1). However, the project targets were not fully reached using this system, specifically with the 1:1 H_2_/N_2_ feed stream. If the MEMPHYS system is to be used on a feed stream with <50% hydrogen content, the electrical power demand or CAPEX will increase significantly. In their conclusion, the authors state some possible adjustments that could be made to the system (e.g., addition of an ozone generator) and their (future) intention to carry out further testing with other substances in the anode gas mixture.

- **H_2_/CH_4_/Ar**

Onda et al. [169] carried out preliminary investigations into the separation and compression efficiencies, and performance of a hydrogen pump and found that >98% of hydrogen in the feed could be separated, at cell voltage 0.06–0.15 V and current efficiencies ~100%. They developed a simulation code, based on a pseudo two-dimensional code for a proton exchange membrane fuel cell (PEM-FC). The current distribution along the flow direction calculated by their simulation agreed well with the measured distribution, except when the H_2_ concentration is low and the H_2_ transport rate is high.

- **H_2_/CH_4_/Ethylene**

Doucet et al. [171] investigates the feasibility of separating hydrogen from H_2_/ethylene gas mixtures. Experimental results showed that a large amount of the ethylene reacted with the H_2_ to form ethane. In spite of this reaction taking place, results still indicated that it is possible to obtain reasonable separation. Results indicated that if Pt is used as the electrocatalyst to separate alkenes and hydrogen, the consumption of hydrogen is somewhat less than the amount of alkenes in the inlet. The suggestion was made that another catalyst (which has a lower hydrogenation rate and different selectivities for H_2_ and ethylene adsorption) should be used when alkenes are present, as it might improve the general efficiency of the process. The authors concluded that EHS by means of PEM-FC technology is not suitable for large quantities of ethylene.

##### “Active” Gas Mixtures

- **CO and CO_2_-Containing Mixtures**

Lee et al. [166] investigated the electrochemical separation of hydrogen from a H_2_/N_2_/CO_2_ mixture. They reported that an increase in cell temperature resulted in enhanced hydrogen purity and energy efficiency. Application of pressure on the feed gas side increased the performance and the amount of hydrogen produced but decreased the hydrogen purity, as the permeation flux of impurities increased. The performance decreased, with a higher concentration of impurities in the feed gas stream. The permeability of CO_2_ is greater than that of N_2_, therefore, the hydrogen purity is more dependent on CO_2_. At a current density of 0.7 A·cm^–2^, a hydrogen purity of 98.6% and 99.73% can be achieved from a low-purity feed (30% H_2_) using a one-stage and two-stage process, respectively.

Gardner and Turner [156] investigated hydrogen separation from hydrogen-rich streams obtained from steam reforming of a hydrocarbon or alcohol source (H_2_/CO_2_ and H_2_/CO_2_/CO gas mixtures). The cell resistance was ~17 mΩ (using pure hydrogen to plot a polarization curve) and pure hydrogen yielded efficiencies of >80%. When extracting hydrogen from a H_2_/CO_2_ mixture, the extraction efficiency was ~80%. However, when the hydrogen feed stream contained CO (1000 ppm), the efficiency was relatively poor. CO severely contaminates the anode, raising the anode potential by up to 300 mV. In attempt to improve the efficiency in the presence of CO, periodic pulsing was examined. It was determined that pulsing can substantially mitigate the problem of CO contamination and reduce the anode potential, although the anode potential is still very high compared with that of an unpoisoned cell. The maximum current densities observed were ~0.2 A·cm^−2^.

Onda et al. [172] measured the voltage–current characteristics of an electrochemical hydrogen pump (EHP) and PEM-FCs; they changed the hydrogen concentration from 99.99% to 1%. Nearly all of the hydrogen could be separated and recovered, even when the hydrogen feed mixture flow rate or feed gas concentration was changed (impurity concentration in the treated gas stream: Max. 1000 ppm). The concentration of hydrogen released at the anode side was ~50 ppm, which is well below the permissible limit (1%). The cell voltage became unstable for CO_2_ gas mixtures

Abdulla et al. [173] investigated the recovery and energy efficiency of hydrogen separation from mixtures of CO_2_, H_2_O and H_2_ using an EHP. Measurements were recorded as functions of the operating parameters: Gas flow rate, gas composition, applied potential difference, and temperature. High-purity hydrogen (>99.99%) was recovered in single-stage EHP experiments, at energy efficiencies of 45%. Experimental analysis revealed that energy efficiencies of >90% with >98% hydrogen recovery is possible with a programmed voltage profile multistage EHP, and 90% hydrogen recovery is achievable with 75% energy efficiency if a fixed applied potential difference multistage EHP is used. The experimental results were used as a basis for predictive models in single-stage and multistage EHPs. 

Wu et al. [160] compared carbon-supported Pt/C and Pd/C catalysts in the electrochemical recovery of hydrogen from H_2_/CO_2_ reformate mixtures. Electrochemical activity and separation efficiency were evaluated using cyclic voltammetry, polarization, and potentiostatic hydrogen pumping. Results revealed that the MEA resistance increased as the dilution of hydrogen in CO_2_ increased. Furthermore, the effective resistance of the Pd/C catalyst was greater than that of the Pt/C catalyst. The CO_2_ adsorbed more strongly to the surface of the Pd/C catalyst, thus impairing the electrochemical active surface area available for hydrogen oxidation/reduction—hence resulting in lower separation efficiencies. Furthermore, the Pd/C catalyst showed lower resistance to CO_2_ poisoning, higher effective ohmic resistance, and a lower mass transport coefficient of hydrogen. The authors concluded that it would be possible to replace Pt with Pd as catalyst to reduce costs; however, this will be accompanied by reduced energy efficiencies. 

Kim et al. [176] investigated the replacement of Pt electrocatalysts with Ir-based electrocatalysts, and carried out the EHS from H_2_/CO_2_ mixtures. The Ir-based catalysts were characterised using X-ray diffraction, X-ray photoelectron spectroscopy, transmission electron microscopy and thermogravimetric analysis. CO_2_ stripping indicated that the Ir catalysts were unaffected by CO_2_, unlike the Pt catalysts. Furthermore, the performance of the Ir catalysts were better than that of the Pt catalysts in terms of H_2_/CO_2_ separation. The operating voltage to separate hydrogen from the CO_2_ mixture was less for Ir300 (0.18 V at a current density of 0.8 A·cm^−2^) than for Pt (0.20 V at a current density of 0.8 A·cm^−2^).

Bouwman [177] reported on electrochemical hydrogen compression and EHS. They used a single cell stack with a pumping capability of 2 kg H_2_/day. Hydrogen was separated from a premixed gas mixture containing 70.05% H_2_, 19.97% CO_2_, 7.477% CO and 2.507% CH_4_. Results revealed that successful hydrogen purification could be achieved with 188 ppm CO_2_, 14 ppm CO, and no detectable CH_4_ in the permeate. The estimated concentration reduction was 1000× for CO_2_, 5000× for CO, and “infinite” for CH_4_.

Chen et al. [178] investigated the possibility of coupling H_2_/CO_2_ separation with hydrogenation of biomass–derived butanone in an EHP, and replacing Nafion membranes with non-fluorinated SPPESK (sulphonated poly (phthalazinone ether sulphone ketone)) membranes. Due to higher resistance, caused by swelling, the SPPESK-based EHP reactor had exceptional reaction rates at elevated temperatures (60 °C). It also had a higher butanone flux of 270 nmol·cm^−2^·s^−1^, compared to that of a Nafion-based EHP reactor, which had a butanone flux of 240 nmol·cm^−2^·s^−1^. Furthermore, the SPPESK-based EHP exhibited better hydrogenation than an EHP with Nafion membranes due to lower CO_2_ permeation; however, efficiencies of the former approximately 20% lower than those of the latter. With H_2_/CO_2_ as feed stream, an efficiency of 40% was reached with the EHP, with a power consumption of 0.3 kWh per Nm^3^ hydrogen. This is superior to what is reached with alternative processes such as PSA and water electrolysis. The separation efficiencies of the membranes used in this study by Chen et al. [178] are tabulated in Table 5. 

Ru et al. [179] conducted a series of experiments at different currents (0–2.5 A), feed flow rates (90 mL·min^−1^–300 mL·min^−1^) and temperatures (25 °C, 50 °C) to determine the optimal operating conditions. A gas mixture of 50:50 H_2_/CO_2_ were fed to the cell at 1 atm and purified. Results showed that the optimal operating conditions were a feed flow rate of 90 mL·min^−1^ at 2.5 A, with 93.62% hydrogen purity and 60.45% hydrogen recovery. The hydrogen purity was enhanced by increasing the temperature from 25 to 50 °C. However, the hydrogen permeation was much lower at 50 °C compared to 25 °C. 

Nordio et al. [180] investigated several parameters of an electrochemical hydrogen compressor/separator, including the hydrogen recovery factor (HRF), hydrogen purity and concentration, the mixture type, the total flow rate, and the temperature. An experimental and modelling (Matlab) approach was applied to study these parameters. The results were promising with regards to HRF and purity. The hydrogen purity, in the case of N_2_ and CH_4_, was ~100%, and more or less >98% in the case of He. Elevating the temperature had a positive effect on hydrogen separation due to increasing membrane conductivity and decreasing ohmic resistance. The model that was developed by these authors was able to effectively predict both the polarization curves and the product hydrogen purity. Finally, a case study in which 75% H_2_:25% CH_4_ and 30% H_2_:70% CH_4_ was used revealed that the EHS is more flexible regarding the hydrogen feed content (lower H_2_ concentration) compared to PSA. 

Nordio et al. [181] investigated the performance of an EHS/compressor for binary H_2_/CO_2_ mixtures. More specifically, they investigated whether the reverse water–gas shift (RWGS) or electrochemical CO_2_ reduction is responsible for CO formation and subsequent catalyst poisoning. They demonstrated that the RWGS is largely responsible for the decreased performance. The lower hydrogen product purity, in comparison with other gas mixtures, was attributed to the extremely high CO_2_ water solubility (in comparison with the other gases). Higher voltages, CO_2_ concentrations, and temperatures have a more pronounced adverse influence on the cells’ performance, together with increased catalyst inhibition. The hydrogen product purity increased with lower CO_2_ concentration in the feed and higher applied voltage. Increasing the temperature resulted in in a promotion of the RWGS reaction, and subsequently led to a more rapid hindrance of the catalyst. Finally, the authors experimentally verified the fast performance of air bleeding, compared to temperature swing desorption—to generate the catalyst.

Jackson et al. [183] investigated the low-loading, high mass transport “floating electrode” technique for EHP catalyst characterisation. The feed streams were pure H_2_ and 20 ppm CO in H_2_. The cyclic voltammograms for Pt/C, Pt-Ru/C and Pt-Ni/C were compared, and the poisoning effects in both in situ and ex situ systems were discussed. Furthermore, the kinetic modelling of CO poisoning on the floating electrode was addressed. The model is based on the Langmuir–Hill isotherm, and takes into account the complex nature of CO_ads_ over time. Result showed that mass activities of 68–93 A·mg_metal_^−1^ were achieved. This is significantly higher than that achieved with the EHP: 6–12 A·mg_metal_^−1^. The authors attributed this to mass transport limitations (water electroosmotic drag). CO poisoning of the EHP and floating electrode systems showed the same tendency in terms of the CO tolerance of the catalyst—Pt-Ru/C>Pt/C>Pt-Ni/C. Finally, the kinetic model was well fitted to the experimental floating electrode data. 

Jackson et al. [184] investigated the operation of an EHP under different EHP poisons. First, the effect of 20 ppm CO was investigated using Pt/C and Pt-Ru/C as anode catalysts. Secondly, a gas mixture, comparable to a product stream from steam methane reforming water–gas shift (SMR-WGS) reactor (78.6% H_2_, 18.5% CO_2_, 2.9% CH_4_ and 20 ppm CO) was tested. Then, lastly, the effects of H_2_S (100 ppb, 1 ppm and 5 ppm in H_2_ and 100 ppb in the SMR-WGS H_2_ feed) was investigated. Two poison mitigation strategies were introduced and investigated: “Online” and “offline” regeneration. This approach included O_3_ in the O_2_ bleed (online cleaning). The ozone was also used to recover the system after exposure to poisons (offline cleaning). It was determined that the gas compositions that contained O_3_ were more effective in cleaning poisons (e.g., *CO_ads_* and *S_ads_*) than the streams containing only O_2_. In the case of severely poisoned streams, the inclusion of O_3_ doubled the achievable current density.

#### 3.2.2. High-Temperature EHS 

##### “Passive” Gas Mixtures

- **H_2_/CH_4_ Mixtures**

Vermaak et al. [188] investigated the EHS from H_2_/CH_4_ mixtures. The article reported on performance parameters such as polarization curves, limiting current density, open-circuit voltage, hydrogen permeability, hydrogen selectivity, hydrogen purity and cell efficiencies (current, voltage and power efficiencies). Three compositions were tested: 10%, 50% and 80% CH_4_ in hydrogen. When compared to the polarization curves of pure hydrogen, under the same operating conditions, these mixtures showed a diluent effect with no affinity towards the membrane or the catalyst. High-purity grade (>99.9%) hydrogen was generated for all three feed compositions. The authors concluded that high-purity hydrogen can be generated from H_2_/CH_4_ mixtures in a single stage process regardless of the methane concentration of the feed. Moreover, the authors found that the hydrogen purity was slightly enhanced with an increase in temperature. High hydrogen selectivities of up to ~22,000 were reached. The hydrogen selectivities were inversely related to temperature. They also reported on hydrogen separation from H_2_/CO_2_ and H_2_/NH_3_ gas mixtures. 

##### “Active” Gas Mixtures

- **CO and CO_2_-Containing mixtures**

Perry et al. [49] investigated and reported on the performance of a HT PBI membrane (processed by the sol-gel process). The electrochemical cell was operated at 120–160 °C with various gas reformate feed streams, with and without humidification. This was done in order to evaluate key parameters, such as the power requirements of the cell, the electrochemical efficiency, the durability, the CO tolerance, and the hydrogen purification efficiency. Almost Faradic flows (correlating to the law of Faraday) were achieved and little power was required to operate the hydrogen pump. In long-term tests, durability was excellent. Polarization curves were constructed to investigate the power requirements. For a typical current density of 0.2 A·cm^−2^, low voltages of ~45 mV were achieved at 160 °C for pure hydrogen. These low voltages are an indication of facile oxidation and reduction of hydrogen and low resistance of the MEA and the cell hardware components. With a stoichiometric hydrogen feed of 1.2 (without humidification), an increase in linearity of the outlet flow rates to current density was recorded.

Thomassen et al. [93] investigated simultaneous hydrogen separation and compression using PBI-based PEM fuel cell technology. The tests were performed using pure hydrogen, N_2_/H_2_ mixtures, and reformate feed gas mixtures containing CO, CO_2_ and CH_4_. The energy efficiency of the compression process was found to be 80–90% (based on the lower heating value of separated hydrogen) at hydrogen fluxes of 5–7 Nm^3^·m^−2^·h^−1^. Furthermore, a CO tolerance of ±1.5% was reported, with a reduction in the other gas components in the separated hydrogen of up to 99%. Stable compression was reported up to 0.65 bar differential pressure over the PBI membrane, with almost no increase in the energy consumption.

Kim et al. [174] investigated the effects of Pt loading on the cell performance of a PBI-based EHP by means of polarization curves. This was paired with an investigation into electrochemical impedance characteristics, utilizing MEAs with various Pt loadings on the anode and the cathode. In addition, the gas separation of H_2_/CO_2_ was investigated, with no humidification, at a cell operating temperature of 160 °C. The cell voltage was reported to be a mere 80 mV at 0.8 A·cm^−2^ for a pure hydrogen feed. This was lower than that achieved with perfluorosulphonic acid (PFSA) membranes at a relative humidity of ≤43%. The cell voltage increased by 72% when the anode Pt loading was decreased from 1.1 mg·cm^−2^ to 0.2 mg·cm^−2^. The effect of various catalyst loadings on the cathode were insignificant. 

Chen et al. [175] investigated PBI-based phosphoric acid (PA)-doped fuel cells under simulated reformate gases with various H_2_, N_2_ and CO concentrations. In the absence of CO, the dilution effect of N_2_ had little to no effect on the performance of the cell. However, CO poisoning increased the charge transfer resistance, which substantially decreased the performance. Experimental results indicated that higher operating temperatures supresses the Pt-CO binding reaction, resulting in improved tolerance towards CO.

Huang et al. [182] prepared, characterised, and tested various PA-doped PBI membranes: *para*-PBI, *m/p*-PBI, and *meta*-PBI. Experimental test also included a conventional imbibed *meta*-PBI, for comparison. Various chemistries of PBI were investigated to understand how the chemistry affected the EHS performance, including the voltage requirement, power consumption, efficiency, hydrogen purity, and also long-term durability of the MEAs. Controlling the chemistry and increasing the polymer solids content led to considerable improvement in the creep resistance of the *m/p*-PBI and *meta*-PBI membranes (<2 × 10^–6^ Pa^–1^) compared with *para*-PBI (~10 × 10^–6^ Pa^–1^). However, the conductivity of *m/p*-PBI and *meta*-PBI exhibited lower proton conductivity (~0.14–0.17 S·cm^−1^) compared with *para*-PBI (0.26 S·cm^−1^). HT PEM cells based on these novel PBI membranes were used to investigate the EHS from various feed gases: Pure hydrogen, and two premixed reformate streams containing H_2_, N_2_ and CO. The reformate streams were used to validate the increased value of this technique when operated at 160–200 °C due to the increased Pt tolerance to CO. Results indicated that the cell can be operated using a dilute hydrogen feed streams with large amounts of CO (1–3%). Fairly pure hydrogen products (>99.6%) were achieved, with high power efficiencies (up to ~72%). 

Vermaak et al. [188] investigated the EHS performance of a TPS membrane (supplied by Advent Technologies Inc., USA) using H_2_/CO_2_ gas mixtures and comparing it to pure hydrogen, H_2_/CH_4_ and H_2_/NH_3_ (refer to the relevant sections). The compositions of the feed mixtures were 10% and 50% CO_2_, balance hydrogen. Compared to pure hydrogen and the H_2_/CH_4_-mixtures, the CO_2_-containing mixtures had a significant effect on the performance of the cell. The polarization curves showed a significant decrease in performance, with a maximum current density of 0.16 A·cm^−2^ for a 100 mL·min^−1^ hydrogen flow rate. The decrease in cell performance could attributed to the reduction of CO_2_ to CO by the RWGS reaction. However, the authors indicated that no CO was detected in the in-line GC, but rather trace amounts of CH_4_, suggesting that CO_2_ methanation might have occurred. In terms of hydrogen purity, 98–99.5% was reached with 10% CO_2_ in the feed and 96–99.5% with 50% CO_2_ in the feed stream. Reasonable separation was achieved with the 1:1 H_2_/CO_2_ mixture, with selectivities of up to ~200.

- **H_2_/NH_3_ Mixtures**

**Figure 5:**
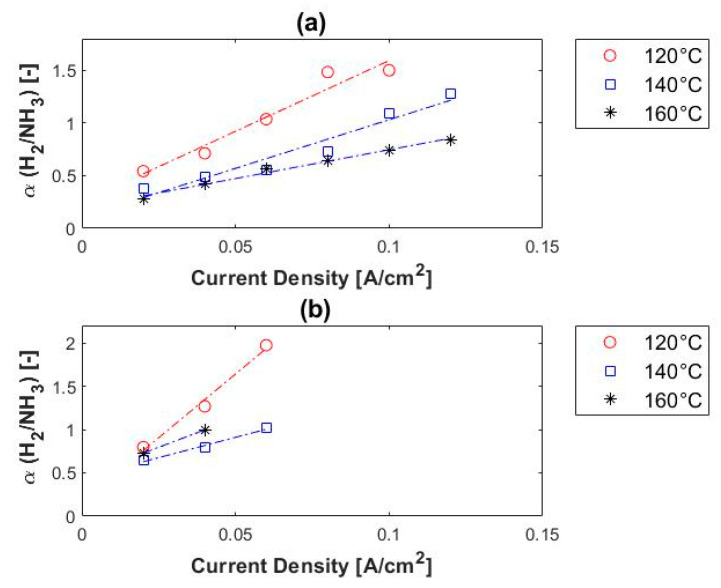
H_2_/NH_3_ selectivity for: (**a**) 1500 ppm and (**b**) 3000 ppm NH_3_ (balance hydrogen) [188].

Vermaak et al. [188] experimentally investigated the separation performance of a EHS system using H_2_/NH_3_ gas mixtures at 120–160 °C. The compositions were 1500 and 3000 ppm NH_3_, respectively, with the balance hydrogen. The cell performance was poor overall. The highest current density that was reached were 0.12 A·cm^−2^. The selectivities were <2 for both feed streams (see Figure 5). The authors reported that NH_3_ and H_2_ competed: In the cases where the selectivity values <1, ammonia transport through the membrane was favoured. Whereas hydrogen transport was favoured at selectivities >1. Ammonia is transferred through the membrane as NH^4+^. The authors reported severe irreversible damage to the membrane and attributed it to the alkaline nature of NH^4+^. They further state that NH^4+^ reacts with the phosphoric acid (PO_4_^3−^) that the membrane is doped with, similar to the reaction seen with Nafion membranes in fuel cells [190,191]. 

#### 3.2.3. Review Articles 

Granite and O’Brien [167] reviewed some novel methods for CO_2_ separation from flue and fuel gas streams, using EHPs, membranes and chemical looping approaches. Whereas, Rhandi et al. [186] reviewed electrochemical hydrogen compression and separation against competing technologies. In their article, the advantages and disadvantages of EHS/compression is highlighted. The review article of Trégaro et al. [187] serves as part 2 of the aforementioned article, where the challenges that EHS face are discussed. Special emphasis is placed on impurities that influence, or degrade, the cell performance, together with electrocatalysts that are commonly used in EHS. 

#### 3.2.4. Overall Summary of the State of Electrochemical Hydrogen Separation 

In general, very few research articles are available on EHS, as this technology is still relatively new. In summary, the following characteristics can be associated with EHS:

- High selectivity;
- sensitivity to catalyst deactivation (e.g., CO deactivation);
- higher tolerance to “active” impurities (e.g., CO and CO_2_) at higher temperatures;
- the hydrogen flux can be controlled by the current and
- simultaneous hydrogen separation and compression is possible.

Very little information is available on failure and degradation mechanisms of the PEMs used in the scope of EHS. However, since fuel cells and electrolyzers are essentially the same technology used for EHS (with different applications), being an electrochemical cell, the degradation mechanisms [192,193] of both these technologies under different operating conditions can be used to partly fill this gap. Some of the phenomena that complicate electrochemical membrane-based processes include the distribution of water in LT application [194], loss of acid in HT PBI membranes [195] and deformation of the membrane and gas diffusion layers by the assembly compression [195,196,197,198]. The properties of HT PBI membranes and LT Nafion membranes have also been compared in [199]. Besides, EHS and compression, fuel cells and electrolyzers, other useful applications for electrochemical cells have been reported: The reduction of CO_2_ [200], and the electrochemical compression of ammonia [201,202,203]. However, these applications are in the very early stages of research. Besides the conventional EHS membranes, e.g., Nafion (LT) and PBI (HT), the use of solid acids have also been reported [204] in steam electrolysis. Typical operating temperatures for these materials are ~200 °C. However, this technology is very new and still require immense research effort. Moreover, in the field of EHS, ceramic proton-conducting membranes are also known, but are still at the earlier development stage [108]. This technology has been briefly discussed in Section 2.3.3.

## 4. Concluding Remarks

There is still much unknown about EHS, largely because the technology is not yet mature. We conclude this overview with mention of the areas that (we consider) require further research in the field of electrochemical hydrogen separation and its further development:

- Little information on component degradation, beside CO catalyst deactivation. However, the degradation studies performed on fuel cells can be used to fill this gap.
- Understanding the life cycle of an electrochemical hydrogen membrane and how an aged membrane’s performance compare to that of a membrane at the beginning of its life.
- Contribution/s of various impurities (considered separately) to the performance parameters. Impurities that commonly accompany hydrogen streams generated from various traditional hydrogen generation methods include CH_4_, O_2_, N_2_, CO, CO_2_, H_2_S, benzene, toluene, xylene and NH_3_. However, as is evident from Table 4, research articles are only available on EHS from mixtures containing CO, N_2_, CO_2_, CH_4_, Ar, ethylene and H_2_O. Furthermore, HT EHS research mainly included reformate gases. Hence, the contribution of respective impurities, separately, on the performance parameters is largely unknown.
- Further research into HT EHS. From Table 4, it is evident that more information is available on LT separation than HT separation. Further research is required to achieve a broader understanding of the expected extent of separation with respect to the various performance parameters—such as limiting currents, hydrogen recovery, selectivities and fluxes.
- One of the advantages that HT membranes present is the possibility of being able to use catalysts such as iron and cobalt. More information on this topic is required in efforts to determine how beneficial this would be—besides only focusing on the cost reduction.
- Fuel cell application. To date, no studies appear to have been conducted to verify the hydrogen purity of EHS product streams for fuel cell application. Such knowledge could be very beneficial, especially when simulated reformate streams are used from industrial hydrogen production systems.
- EHS from industrial hydrogen streams produced from fuel cells (e.g., product streams from steam methane reforming, partial oxidation and gasification of biomass and coal)
- Simultaneous EHS and electrochemical hydrogen compression, together with the process efficiency in terms of hydrogen purity, hydrogen compression, overall efficiency, etc. Specifically, the simultaneous EHS and compression from H_2_/CO_2_ streams, where both the hydrogen stream (permeate) and the carbon dioxide (retentate) is purified and compressed. Such study will be beneficial in terms of hydrogen production and CO_2_ sequestration (i.e., carbon capture and storage, and even carbon capture and utilization).
- Proton-conducting ceramics could be considered a new and upcoming technology and is also part of EHS. The authors suggest that future reviews be done, similar to the one presented, on this topic.

## Figures and Tables

**Table 1:** Summary of hydrogen production processes, their advantages and disadvantages, and technological status.

Method	Advantages	Disadvantages	TML *	PE ** (%)	Cleanness ***	Impurities	References
**Reforming:**							
SMR ^a^	Most developed industrial process, lowest cost, existing infrastructure, high efficiency, best H_2_/CO ratio	Highest air emissions, system is complex, system is sensitive to natural gas quantities. Capital, operation, and maintenance cost. Fossil fuel feedstock.	10	65–75	NC/CCS	CO_2_, CO, CH_4_, N_2_	[2,3,46,55,59,60,61,65,68,75,76,77,78,79]
POX ^c^	Well-established. Variety of fuels, reduced desulphurization requirement, no catalyst required	Complex handling process, high operating temperature, low H_2_/CO ratio. Fossil fuel feedstock	7–9	50	NC	CO, CO_2_, H_2_O, CH_4_, H_2_S, COS and sometimes CH_4_	[46,60,61,65,66,78,80]
ATR ^b^	Lower temperatures than POX ^c^, Requires less oxygen than POX ^c^	Limited commercial application, required air or oxygen. Fossil fuel feedstock.	6–8	60–75	NC	CO, CO_2_, N_2_, CH_4_ and sometimes Ar	[60,81]
**Gasification:**							
Coal	Abundant and affordable, Low-cost synthetic fuel in addition to H_2_	Reactor costs, system efficiency, feedstock impurities, significant carbon footprint unless CCS is used. Separation and purification of gas products are difficult [82]. Fossil fuel feedstock (coal gasification). Season limitations and heterogeneity (biomass)	10	74–85	NC/CCS	N_2_, CO_2_, CO, CH_4_, H_2_S	[79,83,84,85]
Biomass			3 (R&D)	35–50	NC/CCS	CO_x,_ SO_x_ and CH_4_	[2,78,84,86,87]
**Electrolysis:**							
Water electrolysis	Simplicity of process design, compactness, renewable feedstock, cost effective way to produce hydrogen locally. Does not involve moving parts. Silent operation.	Energy input is required and it is more costly than fossil-fuel alternatives.	9–10	62–82	C	H_2_O	[2,66,67]

* Technology maturity level (TML) is defined by a rating scale (1–10) used to indicate the commercial readiness of the technology. Level 1 refers to initial research stages, whilst level 10 refers to well-established mature commercial technologies [2]. ** Process efficiency (PE). *** C = clean without emissions, NC = not clean with emissions, CCS = quasi-clean using carbon capture and storage (CCS). Abbreviations: ^a^ Steam methane reforming (SMR). ^b^ Auto-thermal reforming (ATR). ^c^ Partial oxidation (POX).

**Table 2:** Hydrogen fuel quality specifications.

Constituent	Limits (μmol·mol^−1^ Unless Stated Otherwise)	Minimum Analytical Detection Limit
Hydrogen fuel index	>99.97%	
Water ^a^	5	0.12
Total hydrocarbons ^b^ (C_1_ basis)	2	0.1
Oxygen	5	1
Helium	300	100
Nitrogen, Argon	100	5
Carbon dioxide	2	0.1
Carbon monoxide	0.2	0.01
Total sulphur ^c^	0.004	0.00002
Formaldehyde	0.01	0.01
Formic acid	0.2	0.02
Ammonia	0.1	0.02
Total halogenates ^d^	0.05	0.01
Particulate concentration	1 mg·kg^−1^	0.005 mg·kg^−1^

^a^ Due to the water threshold level, the following should be tested for, if there are concerns regarding the water content: (1) Sodium (Na^+^) < 0.05 μmol·mol^−1^ H_2_ or < 0.05 μg·L^−1^; (2) Potassium (K^+^) < 0.05 μmol·mol^−1^ H_2_ or < 0.08 μg·L^−1^ (3) Pottasium hydroxide (KOH) < 0.05 μmol·mol^−1^ H_2_ or < 0.12 μg·L^−1^; ^b^ e.g., ethylene, propylene, acetylene, benzene, phenol (paraffins, olefins, aromatic compounds, alcohols, aldehydes). The summation of methane, nitrogen and argon is not to exceed 100 ppm. ^c^ e.g., hydrogen sulphide (H_2_S), carbonyl sulphide (COS), carbon disulphide (CS_2_) and mercaptans. ^d^ e.g., hydrogen bromide (HBr), hydrogen chloride (HCl), Chlorine (Cl_2_) and organic halide (R-X).

**Table 3:** Properties of different hydrogen purification processes (adapted from Refs [2,95,98,99]).

Properties	PSA	Membranes	Cryogenic
Min. feed purity (vol.%)	>40	>25	15–80
Product purity (vol.%)	98–99.999	>98	95–99.8
Hydrogen recovery (%)	Up to 90	Up to 99	Up to 98

**Table 4:** Summary of published articles on electrochemical hydrogen separation.

Year	Type	Membrane	Catalyst *	Impurities	Temp. (°C)	Refs.
2004	Experimental	Nafion	Pt (B)	N_2_, CO_2_	30–70	[166]
2005	Review	N/A	N/A	N/A	N/A	[167]
2007	Experimental	Nafion	Pt (A), Ru (C)	CO, CO_2_	20	[156]
	Experimental/modelling	Nafion	Pt/Pt-Ru (B)	Ar, CH_4_	20–70	[168]
	Simulation	N/S **	N/S **	N_2_	25, 60	[169]
2008	Experimental	Nafion	Pt (B)	N_2_	25, 60	[170]
	Experimental	PBI	Pt (B)	CO, CO_2_, N_2_	120–160	[49]
2009	Experimental/modelling	Nafion	Pt (B)	Ar/C_2_H_4_	25	[171]
	Experimental	N/S **	N/S **	N_2_/CO_2_	60	[172]
2010	Experimental	PBI	N/S **	N_2_; CO_2_, CO, CH_4_; N_2_,CO_2_, CO	160–180	[93]
2011	Experimental	Nafion	Pt/C (B)	CO_2_, H_2_O	50–70	[173]
	Experimental	Nafion	Pt (B)	N_2_	35, 55, 75	[161]
	Experimental	Nafion	Pt (B)	Ar	20–70	[159]
2012	Experimental	Nafion	Pt/C, Pd/C (B)	CO_2_ reformate	30–50	[160]
2013	Experimental	PBI	Pt(B)	CO_2_	80, 160	[174]
2014	Experimental	PBI	Pt (B)	Simulated reformate: N_2_, CO	140–160	[175]
	Experimental	Nafion	Ir/C (B)	Ar, CO_2_	25, 70	[176]
2016	Experimental	Nafion	N/S **	CO_2_, CO, CH_4_	25–75	[177]
	Experimental	SPPESK, Nafion	Pt (B)	CO_2_	20–60	[178]
2018	Experimental	Nafion	Pt-Ru (A), Pt (C)	CO_2_	25, 50	[179]
2019	Experimental/modelling	Nafion	N/S **	N_2_, CH_4_, He, CO_2_	≤28	[180]
	Experimental	Nafion	N/S **	N_2_, CO_2_ and air	15–22.5	[181]
	Experimental case study: MEMPHYS (Membrane based purification of hydrogen system) system	N/S **	N/S **	N_2_	35	[97]
2020	Experimental	PBI	Pt (B)	N_2_, CO	160–200	[182]
	Experimental/modelling	Nafion	Pt/C (B), Pt-Ru/C (A) and Pt/C (C), Pt-Ni/C (A) and Pt/C (C)	CO/Ar; N_2_	35	[183]
	Experimental/modelling	Nafion	Pt/C (A), Pt-Ru/C (A) and Pt-Ru(C)	CO; CO_2_, CH_4_, CO, H_2_S	35	[184]
	Case study/modelling	N/A	N/A	H_2_,N_2_ and CO_2_	N/A	[185]
	Review	N/A	N/A	N/A	N/A	[186]
	Review	N/A	N/A	N/A	N/A	[187]
2021	Experimental	TPS	Pt-Co/C (A) and Pt/C (C)	CH_4_, CO_2_, and NH_3_	120–160	[188]

* A = anode; C = cathode, B = both electrodes; ** N/S = Not specified.

**Table 5:** Hydrogen purity of the product gas (inlet stream properties: 56.7 mA·cm^−2^, 40 °C, 1 atm, 75 vol.% CO_2_) [178].

Gas	Composition [%]			
	SPPESK-0.71	Nafion 115	Nafion 212	Nafion/PTFE
H_2_	>99.99	>99.99	99.79	99.25
CO_2_	<0.01	<0.01	0.21	0.75
